# Supplementary material for: MetaRibo-Seq measures translation in microbiomes
Source: Nat Commun. 2020 Jun 29;11:3268. doi: 10.1038/s41467-020-17081-z (PMC7324362; doi:10.1038/s41467-020-17081-z)
Supplement: Supplementary file 10 — Supplementary Data 7 [file 41467_2020_17081_MOESM10_ESM.zip › File2/Confidence_High_Taxonomy/92776_out.krona.html]

Javascript must be enabled to view this page.

members
magnitude
magnitudeUnassigned
count
unassigned
taxon
rank

92776\_out

2

superkingdom
2
2

phylum
1239
2

class
2
186801

2
186802
order

family
2
186803

genus
189330
1

species

SRS024549\_contig\_number\_contig-100\_15126.15126
1262873
1

1
1506577
genus

29361
1

SRS075398\_contig\_number\_87
species
